# Effects of miR-128-3p on Renal Inflammation in a Rat Periodontitis Model

**DOI:** 10.3390/dj13120577

**Published:** 2025-12-03

**Authors:** Mohammad Nurhamim, Yixuan Zhang, Momoko Nakahara, Daiki Fukuhara, Yosei Nagashima, Takayuki Maruyama, Manabu Morita, Daisuke Ekuni

**Affiliations:** 1 Department of Preventive Dentistry, Okayama University Graduate School of Medicine, Dentistry and Pharmaceutical Sciences, Okayama University, Okayama 700-8558, Japan; dr.nurhamim@gmail.com (M.N.); py466nau@s.okayama-u.ac.jp (Y.Z.); pnwd9gis@s.okayama-u.ac.jp (Y.N.); 2 Department of Preventive Dentistry, Faculty of Medicine, Dentistry and Pharmaceutical Sciences, Okayama University, Okayama 700-8558, Japan; pric37ll@s.okayama-u.ac.jp (M.N.); dekuni7@md.okayama-u.ac.jp (D.E.); 3 Department of Preventive Dentistry, Okayama University Hospital, Okayama 700-8558, Japan; silverhappyend@yahoo.co.jp; 4 Advanced Research Center for Oral and Craniofacial Sciences, Okayama University Dental School, Okayama 700-8558, Japan; 5 Department of Oral Health, Takarazuka University of Medical and Health Care, Osaka 531-0071, Japan; m.morita@tumh.ac.jp

**Keywords:** extracellular vesicles, miR-128-3p, mRNA, inflammation, periodontitis, renal inflammation, lipopolysaccharide

## Abstract

**Objectives:** The study aim was to investigate the effects of extracellular vesicles (EVs) derived miR-128-3p on renal inflammation using a rat periodontitis model. **Methods:** Ten-week-old male Wistar rats were divided into two groups: a control (*n* = 8) and a lipopolysaccharides (LPS) group (*n* = 8). The LPS group received LPS (*Porphyromonas gingivalis*) injection in the gingiva for 7 days. At the end of the experiment, plasma, gingival tissue, and kidney samples were collected. Hematoxylin and eosin staining was performed to evaluate the glomerular tissue injury score. Bioinformatic analysis was conducted to identify potential target genes of miR-128-3p. The reverse transcription-quantitative polymerase chain reaction was performed to evaluate miR-128-3p, inflammatory, pro-inflammatory cytokine, chemokine and predicting gene’s expression. The control and LPS groups were compared using Welch’s *t*-test. *p*-values < 0.05 were considered to indicate statistical significance. **Results:** The kidney glomerular tissue injury score was significantly higher in the LPS than in the control group. miR-128-3p expression in the LPS group was significantly higher in the gingival tissue and plasma. mRNAs *(interleukin [IL]-1β, tumor necrosis factor [TNF]-α, C-X3-C motif chemokine ligand 1 [CX3CL1],* and *C-X-C motif chemokine ligand 7 [CXCL7]*) expression was higher in the kidney of the LPS group. The potential target genes of *activin A receptor type I (Acvr1)*, *ribosomal protein S6 kinase B1 (Rps6kb1)*, and *transforming growth factor beta receptor type 1 (Tgfbr1)* were significantly lower in the kidneys of the LPS group. **Conclusions:** EVs-derived miR-128-3p in LPS induced periodontitis may cause kidney inflammation which may be mediated by, *Rps6kb1, Tgfbr1*, and *Acvr1*.

## 1. Introduction

Extracellular vesicles (EVs) are lipid-encapsulated vesicles ranging from approximately 40 to 160 nm in diameter (average ~100 nm) and have the capacity to transport bioactive molecules, including DNA, RNA, lipids, and proteins, to other cells to regulate their biological functions [1]. EVs mediate a complex intercellular communication network in sepsis, shuttling a variety of key mediators, such as microRNAs (miRNAs) [2].

MiRNAs are short noncoding RNA molecules of approximately 21–23 nucleotides in length that negatively regulate gene expression by inhibiting translation or promoting mRNA degradation [3]. Although numerous studies have reported the functional transfer of miRNAs between cells, many of them have provided insufficient evidence to confirm that this transfer is mediated specifically by EVs [4]. Some miRNAs have organ-specific expression and can circulate from one organ to another, potentially changing the expression of specific genes [5], including the promotion or suppression of inflammatory pathways in the kidney [6]. For example, miR-19b-3p is highly expressed in renal tubular epithelial cell-derived EVs and has been shown to mediate macrophage activation by targeting nuclear factor-kappa B (NF-κB)/suppressor of cytokine signaling-1 (SOCS-1) in a lipopolysaccharide (LPS)-induced acute kidney injury (AKI) mouse model [7]. In addition, miR-128-3p has been shown to play a role in inflammation under certain conditions, enhance the inflammatory response, and contribute to sepsis-associated AKI [8]. These findings highlight the crucial role of miRNAs in regulating renal inflammation. However, specific effects of EVs-derived miR-128-3p on renal inflammation remain unclear.

Renal inflammation is the result of a sudden decline in renal function and structural damage caused by various factors, including infections, toxins, ischemia (lack of blood flow), and immune responses [9]. Additionally, renal inflammation is related to the increased production of pro-inflammatory cytokines, chemokines, and adhesion molecules [10], such as tumor necrosis factor (TNF)-α, interleukin (IL)-1, IL-6, IL-8, transforming growth factor (TGF)-β, and regulated upon activation, normal T cell expressed and secreted (RANTES) [11].

Periodontitis is a prevalent inflammatory disease of tooth-supporting tissues characterized by the infiltration of inflammatory cells, disruption of connective tissue attachment, and progressive alveolar bone resorption [12]. It can affect systemic diseases including renal inflammation. One study reported that the periodontitis pathogen *Porphyromonas gingivalis* (*P. gingivalis*) exacerbates acute renal inflammation-induced kidney injury through the secretion of gingipain and the modulation of oncostatin M (OSM) expression, which subsequently disrupts renal tight junctions [13].

On the other hand, miR-128-3p has a significant role in inflammation, and contributes to sepsis-associated AKI [8], liver injury [14], and cholestasis [15]. However, whether miR-128-3p from periodontitis can cause renal inflammation remains unclear.

Given this background, we hypothesized that EVs carrying miR-128-3p derived from periodontitis can induce renal inflammation. To test this hypothesis, we investigated the role of miR-128-3p from EVs associated with periodontitis in renal inflammation and aimed to identify the associated target genes of miR-128-3p in the rat periodontitis model.

## 2. Materials and Methods

### 2.1. Animal and Experimental Design

Male Wistar rats (*n* = 16) aged 10 weeks old (Japan SLC, Inc., Hamamatsu, Japan) were used in this study. All animals were housed in controlled temperature (23–25 °C) and a regular 12 h light/12 h dark cycle. Animals had free access to solid food (MF; Oriental Yeast Co., Ltd., Osaka, Japan) and drinking water ad libitum. Each case contained two rats. The experiment protocol was approved by the Animal Care and Use Committee of Okayama University (approval numbers: OKU-2021669 and 2023774, approval date: 19 December 2023) and followed the Animal Research Reporting of In Vivo Experiments (ARRIVE) guideline.

The rats were randomly divided into two groups: a control group (*n* = 8) and an LPS group (*n* = 8). The rats were allowed to adapt to a new environment for 10 days before any experimental procedures were initiated. The LPS group received LPS derived from *P. gingivalis* (1 mg/mL, InvivoGen, San Diego, CA, USA) [16]. Under inhalation anesthesia 2–4% isoflurane (Viatris Pharma, Tokyo, Japan), a 10 μL injection of LPS was administered between the maxillary first and second molars on both sides of the rats every 2 days for a total of 7 days [16]. The control group received an equivalent volume of distilled water without LPS (Otsuka Pharmaceutical Co., Ltd., Tokyo, Japan) at the same intervals. After the 7-day experimental period, the animals were euthanized under inhalation anesthesia using >4.5% isoflurane (Viatris Pharma) in an induction chamber. The animals were removed from the chamber, and samples were collected immediately.

### 2.2. Sample Collection

Blood samples were collected via cardiac puncture and centrifuged at 12,282× *g* for 30 min to separate plasma from blood using BD Microtainer^®^ K2EDTA tubes (Becton, Dickinson and company, Franklin Lakes, NJ, USA). Gingival tissue from the left maxillary second molar and portions of kidney samples were preserved in RNAlater Solution (Thermo Fisher Scientific, Waltham, MA, USA) for miRNA extraction. The remaining kidney samples were fixed in 4% paraformaldehyde for histological analysis.

### 2.3. Measurement of Alveolar Bone Loss

The right maxillary regions of soft tissue were gently cleaned using curettes and then stained with a 1% aqueous solution of methylene blue (Sigma, St. Louis, MO, USA) for 5 min. Images were captured using a digital camera (Nikon Instruments, Tokyo, Japan). Alveolar bone loss was determined by calculating in millimeters using ImageJ software (version 1.54d; NIH, Bethesda, MD, USA) on the distance between the cementoenamel junction (CEJ), and the alveolar bone crest (ABC) of the second molars (mesial side of the second molar buccal root).

### 2.4. Immunogold Transmission Electron Microscopy (TEM)

TEM was used for checking plasma-derived EVs morphology. Immunogold TEM was performed to check the marker of CD9 and CD63 according to the following steps. Samples were adsorbed onto hydrophilized 100-mesh nickel grids coated with a support membrane and allowed to stand for 15 min. The grids were then blocked with PBS containing 10% goat serum (GEMINI Bio, San Carlos, CA, USA) and 1% BSA for 15 min. Subsequently, sections were incubated with primary antibodies (CD9 and CD63) for 2 h at room temperature, followed by overnight incubation at 4 °C. After washing five times for 5 min each with PBS containing 0.1% BSA, the grids were incubated for 2 h with secondary antibodies (anti-mouse IgG/goat and anti-rabbit IgG/goat; BioLegend, San Diego, CA, USA) conjugated to 10 nm gold colloids (BBI Solutions, Crumlin, UK). The samples were then washed three times for 5 min each with PBS containing 0.1% BSA and fixed in 2% glutaraldehyde for 3 min. After rinsing with distilled water, the grids were stained with uranyl acetate for approximately 30 ss, air-dried, and examined under a transmission electron microscope (H-7650; Hitachi High-Technologies, Tokyo, Japan).

### 2.5. Nanoparticle Tracking Analysis (NTA)

The Zetasizer Nano ZSP (Malvern Panalytical, Malvern, UK) with a 633 nm laser was used to investigate the EV suspension to characterize the number and size of EVs of the isolated plasma EVs. The NTA analysis software (Zetasizer Nano software v3.30; Malvern Panalytical) was used for further analyzing the nanoparticle tracking data based on the videos of completed tracks.

### 2.6. Kidney Histology

Renal tissues were collected from rats and rinsed with 0.1 M phosphate-buffered saline (PBS; pH 7.4). The tissues were then fixed in 4% paraformaldehyde for 24 h. After fixation, the samples were dehydrated through a graded ethanol series (70%, 80%, 90%, and 100%) and embedded in paraffin. Tissue sections were cut at a thickness of 4 μm using a microtome (Yamato Kohki Industry, Saitama, Japan) and subsequently stained with hematoxylin and eosin (H&E) (FUJIFILM Wako Pure Chemical Corporation, Osaka, Japan) for histological analysis. Tissue slides were then checked under a microscope (BZ-X810; Keyence, Itasca, IL, USA) and images were taken for glomerular cell counts. Inflammatory cells (eosinophils and neutrophils) were quantified and analyzed using ImageJ software within the same threshold values [17] in a blinded manner.

### 2.7. Plasma Biochemistry Analysis for Assessing Renal Function

Plasma creatinine and blood urea nitrogen (BUN) levels were analyzed using Fuji Dry-Chem Slide BUN-PIII and Fuji Dry-Chem Slide CRE-PIII on the FUJIFILM Dry-Chem 7000V analyzer (FUJIFILM Corporation, Tokyo, Japan) according to the manufacturer’s instructions.

### 2.8. Detection of Plasma LPS

The LPS level of endotoxin units (EU) was detected in the blood plasma of both groups. The concentration of ≤0.1 EU/mL was measured using the ToxinSensor Chromogenic LAL Endotoxin Assay kit (GeneScript USA, Piscataway, NJ, USA) according to the manufacturer’s instructions. Four endotoxin standard concentrations (0.1, 0.05, 0.025, and 0.01 EU/mL) were used to create the standard curve, where the R^2^ value was 0.9892.

### 2.9. Total RNA Extraction

Total RNA, including miRNA, was isolated from plasma circulating EVs using the Exosomal RNA Purification Mini Kit (Norgen Biotek, Thorold, ON, Canada). Kidney and gingival tissue samples were homogenized with liquid nitrogen using a frozen cell crusher (Microtec, Chiba, Japan) and small RNA extraction was performed using the mirVana miRNA Isolation Kit (Thermo Fisher Scientific) following the manufacturer’s protocol. The quality of the extracted RNA was evaluated by measuring absorbance at 260 nm, while RNA purity was determined by calculating the 260/280 nm absorbance ratio using a UV spectrophotometer (Beckman Coulter, Tokyo, Japan).

### 2.10. The Reverse Transcription-Quantitative Polymerase Chain Reaction (RT-qPCR) of MiR-128-3p

A primer of miR-128-3p (Assay ID: 002216) and U6 (Assay ID: 001973), utilized as an internal control from TaqMan MicroRNA Assays (Thermo Fisher Scientific). For reverse transcription reactions on gingival, kidney, and plasma samples, the TaqMan MicroRNA Reverse Transcription Kit (4366596; Thermo Fisher Scientific) was used. The RT-qPCR reactions were carried out using TaqMan Universal Master Mix II without uracil-DNA N-glycosylase (UNG) (4440043; Thermo Fisher Scientific). The thermal cycling conditions included UNG activation at 50 °C for one cycle and enzyme activation at 95 °C for one cycle, followed by 40 cycles of denaturation and annealing at 95 °C for 15 s and 60 °C for 60 s. All steps were performed twice. To determine expression levels (fold-change), we used the 2^(^ΔΔ^Ct) method.

### 2.11. Bioinformatics Analysis

To identify the target genes regulated by miR-128-3p, potential targets were predicted using publicly available algorithms, including miRWalk (http://mirwalk.umm.uni-heidelberg.de/) (accessed on 18 September 2024), TargetScan (Release 8.0) (accessed on 19 September 2024), and miRDB (https://mirdb.org/mirdb/index.html) (accessed on 19 September 2024). Forty-five target mRNAs were identified, each present in at least two databases. To explore their functional annotations, Kyoto Encyclopedia of Genes and Genomes (KEGG) pathway enrichment analysis was conducted using the Database for Annotation, Visualization, and Integrated Discovery (https://david.ncifcrf.gov/) (accessed on 20 September 2024). After inputting the 45 mRNAs, a functional annotation chart of KEGG pathways was generated. The analysis revealed that only the TGF-β signaling pathway was enriched, with three associated mRNAs: *activin A receptor type 1* (*Acvr1*), *ribosomal protein S6 kinase B1* (*Rps6kb1*), and *transforming growth factor beta receptor 1* (*Tgfbr1*).

### 2.12. Primer Design and mRNA Quantification

Primers for *Rps6kb1*, *Tgfbr1*, *Acvr1*, and inflammatory genes (*cyclooxygenase* [*COX*] *2*) [18], pro-inflammatory cytokine genes (*IL-1β* and *TNF-α*) [19], and chemokine genes (*C-X3-C motif chemokine ligand 1 [CX3CL1], C-X-C motif chemokine ligand 7 [CXCL7]*, and *C-C motif chemokine ligand 5* [*CCL5*]) [20] were designed using the National Center for Biotechnology Information (NCBI) database (https://www.ncbi.nlm.nih.gov/) (accessed on 8 October 2024). The *glyceraldehyde 3-phosphate dehydrogenase* (*GAPDH*) gene served as internal control.

Primer specificity was verified from Primer-BLAST using the NCBI database (https://www.ncbi.nlm.nih.gov/tools/primer-blast/index.cgi) (accessed on 8 October 2024). Table 1 shows the list of primer sequences used in this study. To obtain cDNA from the total RNA of kidney, samples were reverse-transcribed using the PrimeScript RT Reagent Kit (TAKARA Bio, Shiga, Japan). The reaction started at 37 °C for 15 min, followed by heating at 85 °C for 5 s to stop the reaction. RT-qPCR was then performed using SYBR Green Real-Time PCR Master Mix (TOYOBO, Osaka, Japan) on a Roche LightCycler 96 (Roche Diagnostics, Basel, Switzerland). The process started with pre-denaturation at 95 °C for 30 s, followed by 40 cycles of three steps: denaturation at 95 °C for 5 s, annealing temperature for 10 s, and extension at 72 °C for 15 s. The relative mRNA expression levels (fold-change) were determined using the 2^(^ΔΔ^Ct) method, with *GAPDH* mRNA used as the reference gene for normalization.

### 2.13. Statistical Analysis

All statistical analyses were performed using GraphPad Prism 8 (Version 8.0.2; GraphPad Software Inc., San Diego, CA, USA). The control and LPS groups were compared using Welch’s *t*-test. Data were presented as the mean ± standard deviation (SD). *p*-values < 0.05 were considered to indicate statistical significance. In mRNAs, to control for multiple comparisons, *p*-values were adjusted using the Benjamini–Hochberg false discovery rate (FDR) procedure and reported as *q*-values. An FDR threshold of Q = 0.20 was used as significant. The effect size was also assessed using Cohen’s d [21]. The small, medium and large effect sizes are d = 0.20, 0.50, and 0.80, respectively.

Sample size was estimated assuming an effect size 0.6 mm (SD, 0.4) of alveolar bone loss based on our preliminary study and a previous report [22]. Based on the data, it was determined that 8 rats per group would be necessary to provide an 80% power with an alpha of 0.05, 2-tailed and *t* test.

## 3. Results

### 3.1. Body Weight

Before euthanizing the animal, the body weight of rats was measured, and no notable difference was found in the weights among the rats. The mean ± SD body weights of rats in the control and LPS groups were 335.25 ± 4.37 g and 336 ± 7.37 g, respectively, with no significant difference observed (*p* = 0.404, d = 0.127).

### 3.2. Alveolar Bone Loss

**Figure 1:**
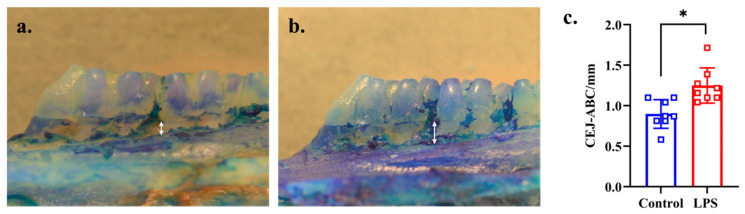
Progression of alveolar bone loss in the control and LPS groups. The white arrow shows the distance from CEJ to ABC of the mesial side of buccal root of second molar. (**a**) Representative image of the control group (*n* = 8). (**b**) Representative image of the LPS group (*n* = 8). (**c**) Comparison of the distance from CEJ to ABC between the control and LPS groups. * *p* < 0.05 compared with the control group (Welch’s *t*-test).

Alveolar bone loss (from CEJ to ABC of the second molars) was significantly greater in the LPS than in the control group (*p* = 0.002, d = 1.330), as shown in Figure 1a–c.

### 3.3. Characteristics of Plasma EVs

**Figure 2:**
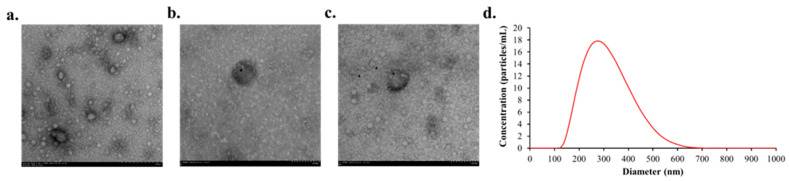
Representative image of characteristics of EVs after isolating them from plasma. (**a**) Representative immunogold TEM results of plasma EVs (**b**) Primary antibody marker CD9 attached to the EVs identified in TEM (**c**) Presence of CD63 marker attached to the EVs (**d**) NTA analysis of plasma EVs.

TEM revealed that the isolated plasma EVs exhibited the characteristic cup-shaped morphology (Figure 2a). Immunogold TEM confirmed the presence of the EV surface markers CD9 (Figure 2b) and CD63 (Figure 2c). NTA showed that the mean diameter of the isolated EVs was 308.6 nm (Figure 2d).

### 3.4. Histological Findings of Renal Tissue

**Figure 3:**
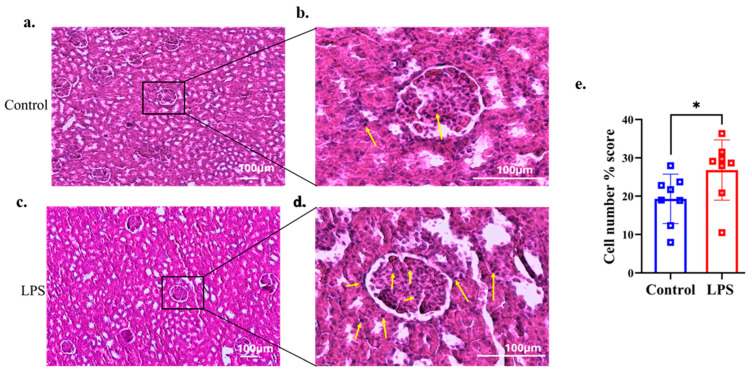
Representative histological images of the renal tissues were stained with hematoxylin and eosin. Yellow arrows show the presence of inflammatory cells (eosinophils and neutrophils). Histological images of the kidney glomeruli of the control group (*n* = 8) (**a**,**b**). The LPS group (*n* = 8) of the kidney glomeruli are shown in (**c**,**d**). (**e**) Percentage scores of inflammatory cells were quantified and analyzed using ImageJ software with the same threshold values in both groups. Data are presented as the mean ± SD and dot plots. * *p* < 0.05 compared with the control group (Welch’s *t*-test).

Histological analysis using H&E staining revealed a higher infiltration of inflammatory cells (eosinophils and neutrophils) in the glomerular area of renal tissues in the LPS group compared with the control group, as shown in Figure 3a–d. Percentage scores of inflammatory cells in the renal tissue were analyzed in both groups. The LPS group showed significantly higher percentage scores of inflammatory cells than the control group (*p* = 0.027, d = 1.231) (Figure 3e).

### 3.5. Plasma Renal Function Analysis

**Figure 4:**
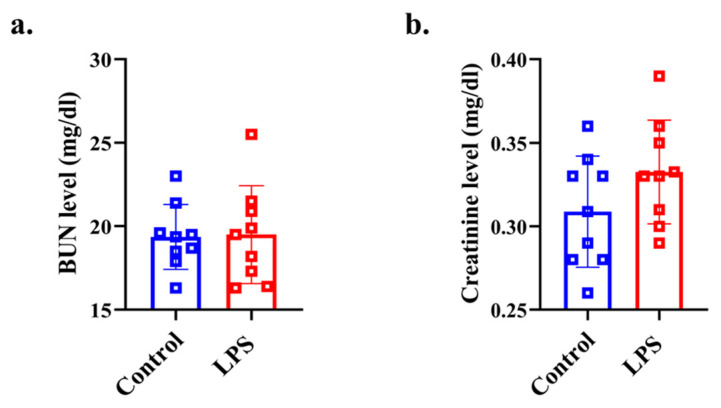
Plasma biochemistry analysis for assessing renal function. (**a**) plasma BUN level, (**b**) plasma creatinine level data from the control (*n* = 8) and LPS (*n* = 8) groups are shown as mean ± SD and dot plots. Statistical significance was calculated using Welch’s *t*-test.

There was no significant difference in the BUN level between the two groups (*p* = 0.459, d = 0.053) (Figure 4a). There was no significant difference in the creatinine level between the two groups (*p* = 0.095, d = 0.669) (Figure 4b).

### 3.6. LPS Level of EU in Blood Plasma

The LPS concentration in blood plasma was measured to investigate the direct effect of LPS on the kidney. The mean ± SD LPS concentrations in blood plasma in the control and LPS groups were 0.00254 ± 0.0007 EU/mL and 0.00250 ± 0.0004 EU/mL, respectively, with no significant difference observed (*p* = 0.451, d = 0.07).

### 3.7. RT-qPCR of MiR-128-3p and Associated Genes

**Figure 5:**
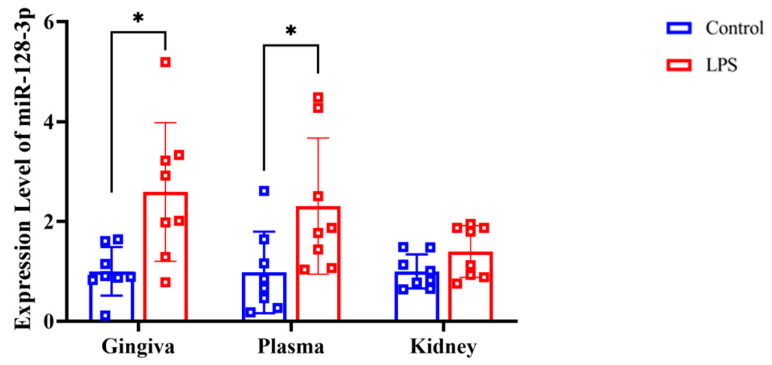
Expression level of miR-128-3p in rat gingiva, extracellular vesicles in the plasma, and kidney tissue as assessed by RT-qPCR (normalized against *U6*). Data from the control (*n* = 8) and LPS (*n* = 8) groups are shown as mean ± SD and dot plots. Statistical significance was calculated using Welch’s *t*-test. * *p* < 0.05 compared with the control group.

RT-qPCR analysis revealed that the expression of miR-128-3p was significantly higher in the gingival tissue (*p* = 0.007 d = 1.224) and plasma (*p* = 0.019, d = 1.033) of the LPS group compared with the control group (Figure 5). However, there were no significant differences in miR-128-3p expression in the renal tissues between the LPS and control groups (*p* = 0.054, d = 0.842).

**Figure 6:**
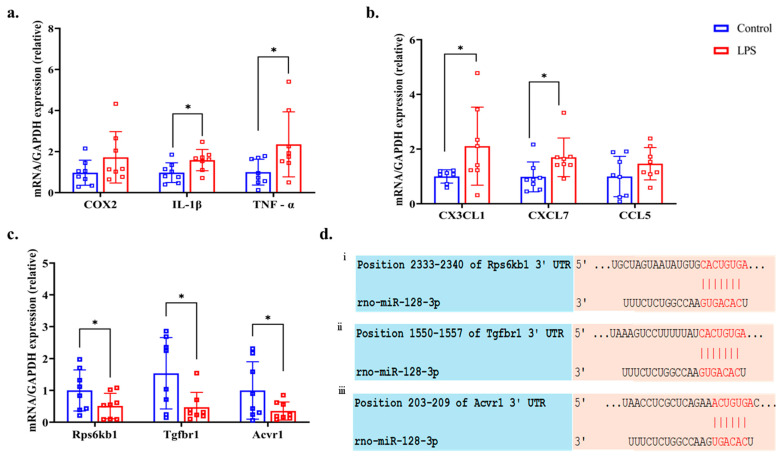
Expression levels of (**a**) inflammatory gene *cyclooxygenase 2* (*COX2*) and proinflammatory cytokine *interleukin* (*IL*)*-1β*, *tumor necrosis factor* (*TNF*)*-α*, (**b**) pro-inflammatory chemokines *C-X3-C motif chemokine ligand 1* (*CX3CL1*), *C-X-C motif chemokine ligand 7* (*CXCL7*), and *chemokine ligand 5* (*CCL5*), (**c**) predictor gene *ribosomal protein S6 kinase B1* (*Rps6kb1*)*, transforming growth factor beta receptor type 1* (*Tgfbr1*)*, and activin A receptor type I* (*Acvr1*) of kidney tissue assessed by RT-qPCR (normalized against glyceraldehyde-3-phosphate dehydrogenase (*GAPDH*) mRNA, and (**d**) predicted binding sites of miR-128-3p and (**i**) *Rps6kb1*, (**ii**) *Tgfbr1*, and (**iii**) *Acvr1.* * *p* < 0.05 compared with the control group (Welch’s *t*-test).

Figure 6a shows the expression levels of the inflammatory gene *COX2* and the pro-inflammatory cytokine genes *IL-1β* and *TNF-α* in the LPS and control groups. Among these genes, *IL-1β* (*p* = 0.034, *q* = 0.076, d = 0.963) and *TNF-α* (*p* = 0.026, *q* = 0.234, d = 0.996) were significantly upregulated in the LPS group, whereas *COX2* (*p* = 0.092, *q* = 0.103, d = 0.685) did not show a statistically significant difference. Additionally, the expression levels of three pro-inflammatory chemokines—*CX3CL1*, *CXCL7*, and *CCL5*—were elevated in the kidneys of the LPS group, as shown in Figure 6b. Among them, *CX3CL1* (*p* = 0.032, *q* = 0.096, d = 0.946) and *CXCL7* (*p* = 0.039, *q* = 0.0702, d = 1.003) showed statistically significant increases, whereas *CCL5* (*p* = 0.103 *q* = 0.103, d = 0.678) did not show a significant difference between the LPS and control group.

### 3.8. Bioinformatics and RT-qPCR of Predicting Genes

From the database, KEGG pathway analysis confirmed that through the TGF-β signaling pathway, the mRNAs- *Rps6kb1, Tgfbr1,* and *Acvr1* had a high probability of being targeted by miR-128-3p at their 3′-UTR regions (Figure 6d). Additionally, RT-qPCR analysis demonstrated that the expression levels of these three predicted target mRNAs—*Rps6kb1* (*p* = 0.046, *q* = 0.059, d = 0.848), *Tgfbr1* (*p* = 0.031, *q* = 0.139, d = 1.070), and *Acvr1* (*p* = 0.044, *q* = 0.066, d = 0.889)—were significantly downregulated in the LPS group compared to the control group (Figure 6c).

## 4. Discussion

We added the new finding that EVs derived miR-128-3p in the periodontitis model contributed to renal inflammation. The results indicated that LPS-induced periodontitis caused overexpression of miR-128-3p in gingiva and EVs in plasma. Higher infiltration of inflammatory cells in the glomerular area of renal tissues in the LPS group were observed. The inflammatory gene *COX2* and pro-inflammatory cytokine and chemokine genes *IL-1β, TNF-α, CX3CL1, CXCL7*, and *CCL5* were upregulated in the kidney. In addition, target genes (*Acvr1, Rps6kb1,* and *Tgfbr1*) of miR-128-3p were significantly downregulated in the LPS group compared with the control group.

EV-derived miRNAs can modulate gene expression in recipient cells, influencing various physiological and pathological processes [23]. miR-128-3p has been shown to play a role in various inflammatory reactions. For example, by regulating miR-128-3p, selenomethionine alleviates myocardial inflammation induced by LPS through the p38 mitogen-activated protein kinase (MAPK) and NF-κB pathways [18]. Inherently, NF-κB is a major regulator of inflammatory responses implicated in the pathogenesis of several diseases [24]. Additionally, miR-128 mediates endotoxin through modulation of the p38 MAPK signaling pathway and causes periodontal tissue damage and alveolar bone loss by initiating excessive inflammation [25]. In the present study, the expression levels of miR-128-3p in gingival tissue and blood plasma were significantly higher in the LPS group, suggesting that miR-128-3p may originate from EVs derived LPS-induced periodontitis.

There were no significant differences in miR-128-3p expression levels of renal tissue between the LPS and control groups. However, there was a large effect (d = 0.842). Furthermore, bioinformatics analysis revealed that the predicted target genes of miR-128-3p were significantly downregulated in the kidney. Thus, the findings suggest that miR-128-3p may reach the renal tissue.

There were no significant differences in BUN and creatinine levels of plasma between the LPS and control groups. The findings suggest that miR-128-3p does not affect renal function in this short-period model. Further long-term experiments will be required to investigate the effects on the function.

The concentration of LPS level of EU in the bloodstream was very low and no significant difference was found between the two groups. This finding suggests that LPS from gingiva did not reach the kidney via the bloodstream, and the mechanism is different with the LPS-induced AKI mouse model [7].

Studies have shown that miR-128-3p promotes kidney inflammation [8,20,26]. For instance, it has been reported that miR-128 promotes inflammation by increasing the expression of inflammatory genes (*Cox2*, *Nfkb*, and *Sod1*) in normal rat kidney epithelial cells [20]. Another study found that miR-128-3p increased the expression of inflammatory cytokines such as TNF-α, IL-1β, and IL-6 [8], whereas IL-1β is a key cytokine in kidney inflammation [26]. Moreover, in the present study, cytokine genes (*IL-1β* and *TNF-α*) expression was statistically significant in the kidney of LPS group and upregulating expression of inflammatory gene *Cox2* was found in the kidney. Based on the findings of the present study and previous studies, EVs derived miR-128-3p may influence the upregulation of *IL-1β* and *TNF-α*, thereby promoting acute inflammation in the kidney. To seek the relationship between periodontitis and renal inflammation, miR-128-3p might be a possible biomarker in clinical studies.

It is also known that kidney cells produce chemokines from the C–C and C–X–C families. During glomerular inflammation in the kidney, CCL5 (RANTES) and CX3CL1 (fractalkine) play a vital role in the infiltration of leukocytes to the site of injury or inflammation [27]. Endothelial cells are likely the source of *CX3CL1* mRNA overexpression in acute renal inflammation in humans [28]. However, activation of rat aortic endothelial cells by LPS and the pro-inflammatory cytokines IL-1β and TNF-α has been shown to result in the strong induction of *CX3CL1* gene expression [29,30]. In the present study, the chemokines *CX3CL1* and *CXCL7* were significantly upregulated in the LPS compared with the control group. The upregulated expression of *CXCL7* and *CCL5* was shown in chemokine array analysis after stimulation of pro-inflammatory cytokines TNF-α and IL-1β [31]. Therefore, it can be assumed that EV-derived miR-128-3p originating from the gingiva, and circulation in the blood may contribute to acute kidney inflammation by promoting the production of *IL-1β* and *TNF-α* in the kidney. Additionally, in the present study, *IL-1β* and *TNF-α* may have been responsible for inducing the chemokines *CX3CL1* and *CXCL7* in the kidney. However, further investigations are needed to elucidate the underlying mechanisms.

After bioinformatic analysis, we confirmed, only the TGF-β signaling pathway was associated with three mRNAs (*Rps6kb1*, *Tgfbr1*, and *Acvr1*) as a potential target gene of miR-128-3p. In this study, we observed that the changes in the expression of *Rps6kb1, Tgfbr1,* and *Acvr1* were significantly downregulated in the kidney. TGF-β1 is widely recognized for its role as an anti-inflammatory cytokine, particularly in facilitating renal repair during the early stages of kidney injury [32]. Dysregulation of TGF-β signaling can lead to increased inflammation and fibrosis in the kidney, contributing to the progression of chronic kidney disease [33]. ACVR1 is a receptor in the TGF-β superfamily that plays a significant role in inflammation and fibrosis. *Rps6kb1* deletion has been shown to reduce the inflammatory senescence-associated secretory phenotype in aged mouse livers, which is characterized by the decreased production of pro-inflammatory cytokines such as IL-1β [34]. Additionally, ribosomal S6 kinase 1 (S6K1) deficiency enhances inflammatory cytokine production [35]. Therefore, findings from the previous studies and this study postulate that the downregulation of potential miR-128-3p target genes (*Rps6kb1*, *Tgfbr1*, and *Acvr1*) may play a role in promoting inflammation in the kidney through the TGF-β signaling pathway.

This study has some limitations. First, because we focused on short-term effects in our acute periodontitis model based on burst theory, the study period was only 1 week [36]. Thus, the long-term effects on the kidney warrant further investigation. Second, we could not investigate the expression of miR-128-3p in the kidney in the other days after LPS administration in the gingiva. The peak of miR-128-3p expression in the kidney might be earlier. miR-128-3p may have become less stable and degraded due to the production of the predicting genes. Third, periodontitis was induced by administering LPS derived from *P. gingivalis*. LPS injection elicits a robust, largely Toll-like receptor 4-driven, non-specific response [37], whereas periodontitis is predominantly T cell-mediated [38]. Differences in the pathogenesis of periodontitis may have influenced the results of this study. Fourth, we have not performed other functional assays or protein measurements in the kidney tissue. Therefore, the induction of AKI in our model remains unclear and requires further study. Finally, male rats were used in this study. Several animal studies suggest that there are substantial gender differences in both normal renal function and the ability to respond to kidney injury [39]. Animal studies suggest that female rats exhibit less kidney injury, inflammation, and interstitial fibrosis than male rats in response to AKI. The generalizability of the results may thus be limited.

## 5. Conclusions

EV-derived miR-128-3p from LPS-induced periodontitis may cause renal inflammation and be mediated by the *Rps6kb1*, *Tgfbr1*, and *Acvr1* genes through the TGF-β signaling pathway. Our findings could be used for further study in the context of renal inflammation linked to periodontitis.

## Figures and Tables

**Table 1:** Primer sequences used in this study.

Name of Genes	Annealing Temp (°C)	Length (bp)	Forward (5′-3′)	Reverse (5′-3′)
*CX3CL1*	67	150	CCCATCCTCATACCCACCTTC	CCACCTGCTCTGTCTGTCTC
*CXCL7*	67	150	CCCCTATTCTTTGTCCTGCTCTT	GGCACACTTCCTTTCCATTCTTC
*CCL5*	66	121	GCTTTGCCTACCTCTCCCTC	GCACACACTTGGCGGTTC
*COX2*	66	131	GGAGGAGAAGTGGGGTTTAGG	TTGATGGTGGCTGTCTTGGT
*TNF-α*	68	133	GGCGTGTTCATCCGTTCTCT	CCCAGAGCCACAATTCCCTT
*IL-1β*	67	145	TGTTTCCCTCCCTGCCTCT	TCATCCCATACACACGGACAAC
*RPS6KB1*	67	140	GTGGATTGGTGGAGTCTGGG	GCTTCTTGTGTGAGGTAGGGAG
*TGFBR1*	68	150	CTGCTTCTCATCGTGTTGGTG	AACTTTGTCTGTGGTCTCGGT
*ACVR1*	67	146	AAGGGCTCATCACCACCAAC	CTTCCCGACACACTCCAACA
*GAPDH*	68	100	ACAAGATGGTGAAGGTCGGT	TCGTGGTTCACACCCATCAC

## Data Availability

The data that support the findings of this study are available from the corresponding author (T.M.) upon reasonable request.

## Abbreviations

The following abbreviations are used in this manuscript:

EVs	extracellular vesicles
miRNAs	microRNAs
NF-κB	nuclear factor-kappa B
SOCS-1	suppressor of cytokine signaling-1
LPS	lipopolysaccharides
AKI	acute kidney injury
TNF	tumor necrosis factor
IL	interleukin
TGF	transforming growth factor
RANTES	regulated upon activation, normal T cell expressed and secreted
OSM	oncostatin M
ARRIVE	Animal Research Reporting of In Vivo Experiments
CEJ	cementoenamel junction
ABC	alveolar bone crest
H&E	hematoxylin and eosin
BUN	blood urea nitrogen
EU	endotoxin units
TEM	transmission electron microscopy
NTA	nanoparticle tracking analysis
RT-qPCR	reverse transcription-quantitative polymerase chain reaction
UNG	uracil-DNA N-glycosylase
KEGG	Kyoto Encyclopedia of Genes and Genomes
Acvr1	activin A receptor type 1
Rps6kb1	ribosomal protein S6 kinase B1
Tgfbr1	transforming growth factor beta receptor 1
COX	cyclooxygenase
CX3CL1	C-X3-C motif chemokine ligand 1
CXCL7	C-X-C motif chemokine ligand 7
CCL5	C-C motif chemokine ligand 5
NCBI	National Center for Biotechnology Information
GAPDH	glyceraldehyde 3-phosphate dehydrogenase
SD	standard deviation
MAPK	mitogen-activated protein kinase
S6K1	S6 kinase 1
